# A Biologically-Based Computational Approach to Drug Repurposing for Anthrax Infection

**DOI:** 10.3390/toxins9030099

**Published:** 2017-03-10

**Authors:** Jane P. F. Bai, Theodore Sakellaropoulos, Leonidas G. Alexopoulos

**Affiliations:** 1 Office of Clinical Pharmacology, Center for Drug Evaluation and Research, U.S. Food and Drug Administration, Silver Spring, MD 20993, USA; teo.sakel@gmail.com; 2 School of Mechanical Engineering, National Technical University of Athens, Zografou 15780, Greece; leo@mail.ntua.gr

**Keywords:** drug repurposing, anthrax, computation, signaling network of a drug

## Abstract

Developing drugs to treat the toxic effects of lethal toxin (LT) and edema toxin (ET) produced by *B. anthracis* is of global interest*.* We utilized a computational approach to score 474 drugs/compounds for their ability to reverse the toxic effects of anthrax toxins. For each toxin or drug/compound, we constructed an activity network by using its differentially expressed genes, molecular targets, and protein interactions. Gene expression profiles of drugs were obtained from the Connectivity Map and those of anthrax toxins in human alveolar macrophages were obtained from the Gene Expression Omnibus. Drug rankings were based on the ability of a drug/compound’s mode of action in the form of a signaling network to reverse the effects of anthrax toxins; literature reports were used to verify the top 10 and bottom 10 drugs/compounds identified. Simvastatin and bepridil with reported in vitro potency for protecting cells from LT and ET toxicities were computationally ranked fourth and eighth. The other top 10 drugs were fenofibrate, dihydroergotamine, cotinine, amantadine, mephenytoin, sotalol, ifosfamide, and mefloquine; literature mining revealed their potential protective effects from LT and ET toxicities. These drugs are worthy of investigation for their therapeutic benefits and might be used in combination with antibiotics for treating *B. anthracis* infection.

## 1. Introduction

The potential use of *B. anthracis* (Gram positive) as a weapon of bioterrorism, combined with recent outbreaks and isolated cases of anthrax infection in the US [1,2] and Europe [3], has focused the developed world’s attention on this lethal bacterium. Notably, the mortality rate during an invasive anthrax infection and the development of shock is exceptionally high when compared to more commonly encountered bacteria [4,5]. Production of LT and ET is closely associated with the pathogenesis of *B. anthracis* infection [6] and the development of shock [7]. LT and ET are both binary-type toxins consisting of protective antigen (the component necessary for host cell uptake of each toxin’s toxic moiety through a membrane anthrax toxin receptor identified in human cells [8]) and lethal factor (LF) for LT and edema factor (EF) for ET [6]. The lethal factor is a metalloproteinase [9] which cleaves and inactivates mitogen-activated protein kinase kinases (MAPKK, including MAPKK’s 1, 2, 3, 4, 6, and 7) [10,11] and also activates host cell inflammasome formation [12,13]. Edema factor has calmodulin dependent adenyl-cyclase activity and increases intracellular cAMP concentrations to high levels [14].

As outlined in Table 1 and Table 2, increasing evidence suggests that both LT and ET target and disrupt the function both of mononuclear and macrophage cells participating in the host innate and adaptive immune responses [15,16,17,18,19,20,21,22] and of endothelial cells maintaining vascular integrity and function [17,23,24,25,26]. While disruption of macrophages by LT and ET is believed to play an important role in propagating early *B. anthracis* infection, disruption of endothelial cell function likely contributes to the highly resistant shock which can develop in some patients with anthrax.

Identifying effective agents that are capable of blocking the pathogenic effects of LT and ET on mononuclear and endothelial cell function could improve the management of this highly lethal infection. To achieve this goal, the present study was designed to utilize reported data regarding the known effects of LT and ET on intracellular molecular targets in either mononuclear or endothelial cells, as well as the mechanisms of action of drugs to identify the already approved drugs that might serve as therapies for anthrax.

**Figure 1:**
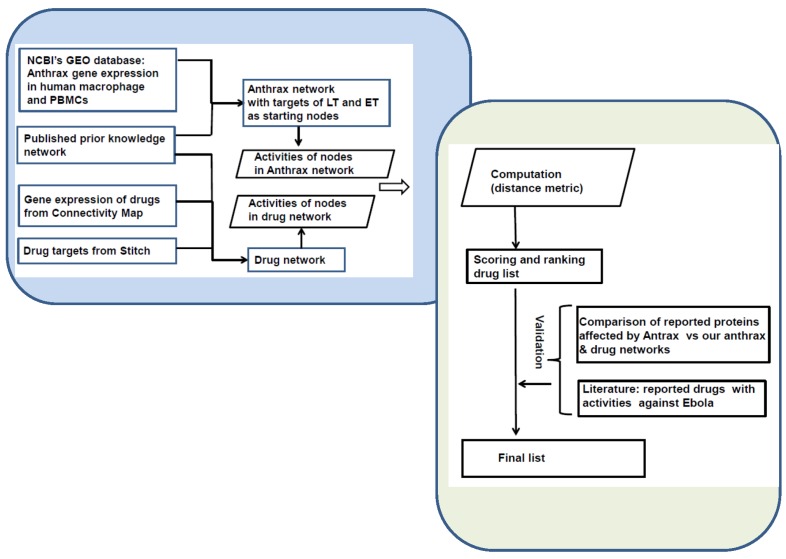
Project workflow: (1) construction of anthrax networks and individual drug networks; (2) computationally scoring individual drugs by computing the distance between anthrax networks and individual drug networks; (3) validation of computed scores and rankings by referencing the literature. We used gene expression data from the Gene Expression Omnibus, prior knowledge network of protein interaction and molecular targets of anthrax toxins in host cells for anthrax networks. Individual drugs’ networks were constructed with gene expression data from the Connectivity Map, prior knowledge network of proteins and their respective targets from STITCH.

We employed our previously published approach [27] to computationally construct the mode of action (signaling network) for each anthrax toxin and drug by utilizing its molecular target(s) and differentially expressed genes, as well as the prior knowledge network (PKN) of protein interactions (Reactome; http://www.reactome.org/). We mined published studies and GEO (Gene Expression Omnibus; http://www.ncbi.nlm.nih.gov/geo/) for the gene expression data generated from human macrophages or peripheral blood mononuclear cells (PBMCs) following exposure to *B. Anthracis* spores or to lethal toxin or to edema toxin. For drugs, we used the differentially expressed genes from the Connectivity Map (CMAP) (https://www.broadinstitute.org/cmap/). We then computationally identified and scored the drugs on their ability to reverse the actions of anthrax’s lethal and edema toxins. To validate our results, we searched the literature to determine whether the top 10 and bottom 10 drugs among the 474 drugs/compounds computationally ranked were supported by literature reports (see Figure 1 for workflow).

## 2. Results

### 2.1. Anthrax Network for Anthrax Toxicity

To construct the signaling network describing anthrax toxicity, we employed our published method [27], where differentially expressed genes induced by anthrax toxins were combined with the prior knowledge network of protein interactions (PKN) which was retrieved from Reactome (http://www.reactome.org/) and the molecular targets of anthrax toxins. The molecular targets of anthrax toxins are MAPKK (mitogen-activated protein kinase kinase) for LT and cAMP for ET, based on publications summarized in Table 1 and Table 2. For the human alveolar macrophage (HAM) dataset, there were 280 significantly perturbed genes (205 up- and 75 downregulated), while for the PBMC dataset there were 407 (309 up- and 98 downregulated) (see Supplementary Materials Table S1, perturbed_genes_cell, consisting of *perturbed_genes_ham & perturbed_genes_pbmc*). Genes that were not present in our PKN [27] or that were not computationally reachable from the known targets of anthrax toxins via the PKN were eventually filtered out from our analysis. The final lists for the two datasets contained 132 genes for the HAM dataset (101 up- and 31 downregulated) and 320 genes for PBMC (45 up- and 275 downregulated) (see Supplementary Materials Table S2, *reactome_genes_cells.txt*). Parts of the PKN not computationally reachable by anthrax targets or not connected with the perturbed gene sets were filtered out.

**Figure 2:**
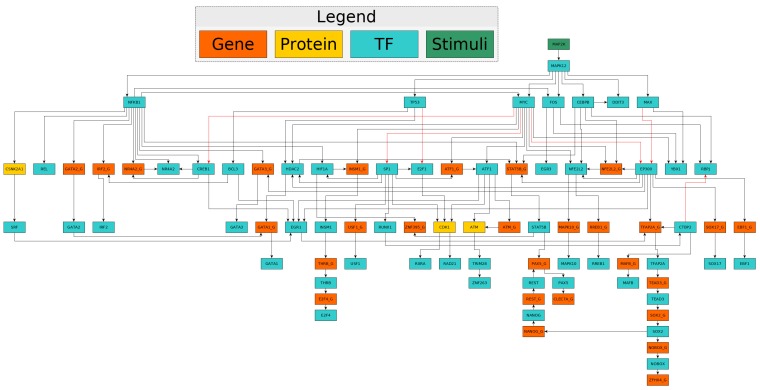
The anthrax network of lethal toxin and edema toxin constructed to reflect the modes of action of anthrax in human macrophages upon exposure to anthrax spores. The gene expression data from the Gene Expression Omnibus (alveolar macrophages that were treated with anthrax spores (GSE14390) were used along with the prior knowledge of protein interaction network (Reactome) and the molecular targets in host cells of lethal and edema toxins).

Because HAM was treated with anthrax spores, we used it as the starting nodes (stimuli) of the signal transduction the targets of both Lethal Toxin (LT), i.e., the MAPKs and NOD-like receptor (NLRP1), and of the Edema Toxin (ET). NLRP1 was reportedly targeted and cleaved by LT, consequently activating inflammasome in rodents [28]. Though the role of NLRP1 in activating inflammasome in human cells is not clearly defined [29], NLRP1 was included because of NLRP1 inflammasome activity in human diseases [30]. Since ET increases the level of cAMP that targets protein kinase A (PKA) [6] and Epac (a RAP1 guanine-nucleotide-exchange factor [31]), we used ET’s secondary targets, i.e., PKA & RAP1 (see Supplementary Materials Table S3, *inputs_cells: inputs_ham & inputs_pbmc*). Considering the GEO database, PBMC cells were treated with LT directly, PKA and RAP1 were left unperturbed in our computation. Since the algorithm was optimized only over the nodes of the network, as a final result we provided only the nodes included in the final network (see Supplementary Table S4, *xs_cells: xs_ham & xs_ham*) as well as the images of the final networks (see Figure 2 for HAM network).

Finally, we compared the retrieved networks with the drug-induced lung injury (DILI) network reported previously [27] where we reported 87 consistently upregulated proteins and 58 downregulated [27]. Out of these, 84 and 36 were also included in our current PKN respectively (two leftmost columns in DILI sheet of see *Results.xlsx*, Supplementary Materials Table S5). We calculated the overlap of our networks with the DILI nodes and computed the probability of this overlap resulting from chance. All the networks were highly enriched in DILI nodes (the *p*-values for HAM-LT, HAM LT-ET and PBMC were 2.47 × 10^−7^, 1.51 × 10^−11^, and 6.23 × 10^−3^, respectively). This analysis was part of our validation of the anthrax network in comparison to our previously published network [27]. Immune cells constitute the defense system circulating throughout the human body and are engaged in all injuries across all organs/systems. Therefore, confirming the extent of overlap between the anthrax and DILI networks served to support the list of drugs that we computationally identified to be potential candidates.

### 2.2. Computation of Network Activities to Identify Candidates for Repurposing

We started out with a list of compounds including approved drugs that were previously studied for drug-induced lung injury [27] and drug candidates profiled on CMAP. The drug targets extracted from STITCH (http://stitch.embl.de/) for individual drugs were compiled. Using the 5% of the top and bottom differentially expressed genes, we identified the upregulated and downregulated genes for drugs profiled on CMAP. We had 652 drugs processed with their individual network activities recovered. In the end, only 474 drugs were included in our computation because some drugs had no connection with the anthrax activity network. We eventually decided to focus on the HAM network when scoring the 474 drugs/compounds instead of combining HAM and PBMC networks to avoid lumping anthrax results from different laboratories. For each drug, we used its network activity and the anthrax activity network in the form of two vectors containing the activities of individual nodes for computation, and scored each drug using Euclidean distance (see *Results.xlsx*, Supplementary Materials Table S5 and 4.3 in Experimental section). The shorter the distance is between the anthrax activity network and a drug/compound’s network, the better the drug/compound’s ability to reverse the anthrax toxicity and the higher its ranking.

We validated our computational results by using a report by Sanchez et al. [32], in which a large number of known drugs were shown to protect cells from anthrax, and other published reports detailed in the section below, and concluded that our computation was reasonable.

### 2.3. Confirmation of Candidates with Reported In Vitro Studies in Literature

We referenced literature and compiled a list of drugs/compounds that have been reported to possess in vitro potency against LT or ET-induced toxicity or increased animal survival. The drugs included bepridil (calcium channel blocker), isotretinoin, sb-203580, propafenone, h-89, and sb-202190 [32], chloroquine [33], niclosamide [34], other calcium channel blockers [35] (verapamil and nitrendipine), simvastatin and fluvastatin [36], MG-132 [37], as well as antibiotics [38,39,40] (doxycycline, ciprofloxacin, chloramphenicol, ampicillin, penicillin, clindamycin, imipenem, vancomycin, clarithromycin, rifampcin, and neomycin). Several other calcium channel blockers that were reported to protect cells from lethal toxin [32] were unfortunately excluded from the list of 474 drugs/compounds owing to lack of gene expression data. Sanchez et al. [32] showed a potency rank order of bepridil > nicardipine > SB-203580 ~ SB-202190 > 13-cis-retinoic acid (isotretinoin) ~ propafenone ~ retinoic acid, all *trans* (tretinoin) ~ H-89. Bepridil, SB-203580, SB-202190, 13-cis-retinoic acid (isotretinoin), propafenone, and H-89 were among the 474 drugs/compounds, with all of them ranked in the top 100 except h-89 (ranked 312) and sb-202190 (ranked 413). Our computation ranked them in the order of bepridil > 13-cis-retinoic acid (isotretinoin) > sb-203580 > propafenone > h-89 > sb-202190. The differences between our ranked order and the potencies by Sanchez et al. could in part be attributed to the fact that they studied cell viability following exposure to LT while our computation considered both LT and ET.

Most interestingly, we ranked bepridil eighth and simvastatin fourth, their top rankings were in agreement with the reports that both drugs were potent in increasing cell survival following exposure to LT [32,36]. The top 10 drugs are listed in Table 3. Notably, literature reports supported their activities for protecting cells from LT or ET toxicity [32,35,36,41,42,43,44,45,46,47,48,49,50,51,52,53]. On the other hand, the bottom 10 drugs listed in Table 4, except monastrol [54], ebselen [55], and genistein [56], had activities similar to those of anthrax toxins, including reduction of MAPK activities or increase of intracellular level of cAMP [32,57,58,59,60,61,62,63]. Monastrol [54] arrested cells in mitosis, unlikely providing protection if cells were under attack by anthrax toxins. As to ebselen [55] and genistein [56], their activities were reportedly opposite to those of anthrax toxins, which would have placed them away from the bottom 10. Our computation might have falsely placed them at bottom 10 or these 2 drugs may have other unknown mechanisms that are harmful to cells.

## 3. Discussion

We computationally ranked 474 drugs/compounds. Among the top 10 drugs, bepridil [32] and simvastatin [36] reportedly increased in vitro cell viability; the other 8 drugs (fenofibrate, dihydroergotamine, cotinine, amantadine, mephenytoin, sotalol, ifosfamide, and mefloquine) had relevant biological evidence to shed light on their potential protective actions (Table 3). However, 7 out of the bottom 10 drugs have actions that are either similar to those of LT and ET or potentially damaging to cells (see details in Table 4). All the nodes in the network had an equal statistical weight and our analysis did not focus any specific nodes. In brief, our computational approach to repurposing drugs for treating anthrax disease has its merit.

Both LT and ET damage endothelial integrity and impair cardiovascular function, leading to fatal shock and tissue injury [64,65]. Fenofibrate is a cholesterol-modifying drug and activates PPARα receptors, and could presumably protect the vascular system from anthrax LT and ET considering PPARα receptors play a key role in endothelial function [66,67]. Most significantly, PPAR α and γ ligands activate MAPKs [68]. Our ranking of fenofibrate among the top 10 drugs supported its direct effect of counteracting the actions of LT and ET at their molecular targets. Simvastatin was reportedly bactericidal, though at a concentration much higher than its therapeutic range observed in humans when used for lowering cholesterol [69]. Several statins increased macrophage viability upon exposure to LT through inhibiting Rho GTPase and activating the MAPK signaling pathways [36]**.** Although simvastatin was ranked in top 10, rankings of other statins beyond 300 by our approach require further investigation.

Ca^+2^ is required for the toxicity of LT [35]. Calcium channel blockers including verapamil and nitrendipine at 100 µM [35] and bepridil [32] at a concentration between 0.125 and 12.5 µM increased cell survival when exposed to LT. Apparently, bepridil was much more potent than verapamil and nitrendipine, and our rankings of bepridil (8th), verapamil (161th), nitrendipine (284th) were in agreement with their reported potencies. Mephenytoin is an active analog of phenytoin that inhibits Ca^+2^ transport into the cells, and our ranking of mephenytoin (6th) was in line with this action [45,46]. On the other hand, ifosfamide, an antineoplastic agent, caused increased renal excretion of Ca^+2^ [52], which could lead to depletion of Ca^+2^ and could thus potentially be protective. . Reportedly, nicotine and its active metabolite, cotinine, activated the mitogen-activated protein kinase pathway [50,51], which is opposite to LT action [10,11]. Paradoxically, cotinine also increased intracellular Ca^+2^ concentration [70], which might enhance LT toxicity. Our ranking of cotinine in the top 10 suggested that its activation of the MAPK pathway at the molecular targets of LT is important.

Sotalol decreased cAMP, which is contrary to the toxic action of ET. Beta blockers including betaxolol, bisoprolol, carvedilol, metoprolol, nadolol, propranolol, sotalol, and timolol decreased cAMP accumulation [53]. Among these drugs, sotalol, metoprolol, and propranolol were among the 474 drugs/compounds ranked in this study. Their reported rank order of potency in reducing the rate of cAMP accumulation was sotalol > metoprolol ~ propranolol [53]. Since ET increased cAMP, the effects of these beta blockers on reducing cAMP accumulation could be beneficial to cell survival. Interestingly, our computation ranked sotalol 9th, metoprolol 13th, and propranolol 109th. Further studies are needed to understand whether they have other mechanisms that might be beneficial.

Dihydroergotamine is a 5-HT 1B/1D agonist [48]; stimulation of 5-HT 1B/1D receptors activated MAPK and reduced cAMP levels [49], shedding light on a possible mechanism of dihydroergotamine in antagonizing anthrax toxins. Our ranking of mefloquine in the top 10 could be linked to the fact that it is an analog of chloroquine and chloroquine reportedly protected cells from LT toxicity [33]. Amantadine reportedly cancelled activation of p38/MAP [44]. Since p38/MAP kinase inhibitors (SB-203580 and SB-202190) protected cells from LT-mediated cytotoxicity [32], such action of amantadine might render protective effects like 38/MAP kinase inhibitors.

The bottom 10 compounds included monastrol, colforsin, berberine, withaferin a, arecoline, ebselen, and genisten. As highlighted in Table 4, berberine [58] and apigenin [62] inhibited MAPK, thereby enhancing LT toxicity. So could withaferin a, arecoline, and beclomethasone. Withaferin a [59,60], arecoline [61], and beclometasone [63] activated p38 MAPK, which is opposite to the action of p38 MAPK inhibitors; inhibitors of p38 MAPK are known to increase cell survival upon exposure to LT [32] (Table 3). Colforsin reportedly increased intracellular cAMP [57], behaving like ET. Monastrol arrested mitosis [54], likely causing harm to cells rather than increasing their survival. As for enilconazole, it is not clear why our computation ranked it among the bottom 10 drugs due to lack of any evidence regarding its effects on Ca^+2^ or cAMP or MAPK. Ebselen activated MPAK p44/42 [55]; so did genisten [56]. Since MPAK p44/42 are downstream of the MAPK pathway; such a downstream activation might not offer any protection, resulting in these two compounds being ranked in the bottom 10 list.

Ciprofloxacin is an antibiotic that has been approved for treating Anthrax disease. The antibiotics in our top 100 drug list included amoxicillin, spectinomycin, ciprofloxacin, alvespimycin, amphotericin b, doxycycline, troleandomycin, benzylpenicillin, and roxithromycin. Since our approach was designed to utilize the modes of action of drugs/compounds at the molecular level for their anti-anthrax activities, the rankings of these antibiotics were indicative of molecular-level interactions between these antibiotics with LT and ET toxic networks in addition to their bactericidal effects on *B. anthracis*.

Heterocyclic small molecules were designed and shown to enzymatically block PA pores and thus protect against anthrax toxin, as reported by Beitzinger et al. [71]. Unfortunately, as our primary goal was to repurpose approved drugs by utilizing gene expression data, knowledge of protein interactions, toxin targets, and drug targets, our approach lacks the ability to explore the effect of inhibiting these pores. Anthrax toxins inhibited chemotaxis of T cells and macrophages resulting in immunosuppression [72,73], and ET promoted development of T_H_2 [74] and T_H_17 cells [75] that are involved in immune responses. Even though chemotactic pathways were not specifically investigated, cytokines, cytokine receptors, and related proteins were included per our computational criteria in our construction of a drug’s activity network and anthrax toxins’ activity networks in macrophages. Interestingly, fenofibrate reportedly preserved expression of CXCR2 on neutrophils and facilitated neutrophil migration, offering protection in sepsis [76]. Simvastatin inhibited IL-5-induced chemotaxis of human eosinophils [77] and reduced C-reactive protein-induced chemotaxis in human monocytes [78]. These reported effects on chemotaxis of both drugs supported their therapeutic potential for treating anthrax infection.

In summary, the extent of agreement of our computed results with literature reports is encouraging. Considering several drugs reported by Sanchez et al. were among our top 100 ranked drugs, the technical merit of our computational approach is worthy of further research. With this said, our computation also took into consideration the effect of ET while Sanchez only focused on LT, likely contributing to the observed differences. The Pearson correlation coefficients between each statin pair were 6.1% for simvastatin (4th) and fluvastatin (62th), 5.3% for simvasatin (4th) and lovastatin (380th), and 2.1% for fluvasatin (62th) and lovastatin (380th). The coefficient was 3.6% between mefloquine (7th) and chloroquine (79th). There seemed to be a weak correlation between Pearson coefficients and our rankings. Among the top 10 drugs, all have been approved by FDA for treating diseases except cotinine. The safety profiles of these drugs are publicly accessible (Drugs@FDA; http://www.accessdata.fda.gov/scripts/cder/daf/). These drugs are worthy of further studies for their clinical utility and optimal dosing in treating anthrax disease, and could be used with antibiotics for the best clinical treatment outcomes if proven therapeutically beneficial.

## 4. Conclusions

Among the 474 drugs/compounds we ranked, the top 10 drugs/compounds have reported in vitro potencies and/or literature evidence to support their biological actions of protecting macrophages from LT and ET toxicities. On the other hand, the bottom 10 drugs/compounds except apigenin, beclometasone, and enilconazole have reported actions similar to those of LT or ET. We ranked simvastatin and bepridil fourth and eighth, which is in agreement with their reported potencies in increasing cell survival. In summary, the top 10 drugs are worthy of investigation for treating *B. anthracis* infection along with antibiotics.

## 5. Experimental Section

### 5.1. Construction of Anthrax Network

Because *B. anthracis* disables the host’s immune defense system and is most deadly if inhaled, we decided to construct an Anthrax’s network using a method published previously [27], where we constructed a drug-induced lung injury network and demonstrated the utility of reversing this network for repurposing drugs for treating lung injury. We applied the published approach [27] and modelled the effect of anthrax infection in the context of a perturbed signaling network where, starting from anthrax’s known targets—i.e., cleavage of MEKs also known as MAPKK (mitogen-activated protein kinase kinase) and upregulation of cAMP (cyclic-AMP)—the signal transduces through the network towards the perturbed genes.

Our strategic approach started with the perturbation of cellular networks by LT of human immune cells, and then used the learned information to identify individual approved drugs with the potential to reverse the toxic signal transduction network of anthrax toxins (LT and ET). The effect of anthrax toxins (LT and ET) was modeled through a perturbed signaling network where, starting from LT’s known targets—i.e., cleavage of MEKs, and ET via up-regulation of cAMP—the signal transduced through a prior knowledge network of protein/protein interactions toward the perturbed genes, as published previously [27]. The activity of anthrax toxins is simply presented with the signs (positive, zero, or negative) induced in every node of the network by the anthrax stimulation. The signs of ‘positive’, ‘zero’, and ‘negative’ represented up-, no, and downregulation of a node, respectively. In order to redirect approved drugs to treat anthrax disease, we simply compared the activity of the drug network to that of anthrax and prioritized the drugs according to the rank order of their ability to cancel out anthrax activity.

We searched GEO for publicly available gene expression data of cells treated with anthrax. We found six experiments of human cells treated with anthrax of any form. In particular, the GEO datasets were:

1. GSE14390: Alveolar Macrophages treated with Anthrax Spores
2. GSE34407: Peripheral Monocytes treated with Lethal Toxin
3. GSE12131: Umbilical Vein Endothelial Cells treated with Lethal Toxin
4. GSE17777: Microvascular Endothelial Cells treated with Edema Toxin
5. GSE4478: Peripheral Monocytes treated with Lethal Toxin
6. GSE12533: Peripheral Monocytes treated with Protective Antigen

For our purposes, we used the human alveolar macrophage (HAM) dataset to rank drug candidates; however, for the purpose of reference and comparison we also analyzed the combined Peripheral Blood Monocytes (PBMC) datasets (these terms represented both types of datasets hereafter). For each dataset, we used the differentially expressed genes published by authors (HAM-LT-ET by Dozmorov et al. [79] and PBMC exposed to LT by Chauncey et al. [80]) and reported in the GEO (Gene Expression Omnibus; http://www.ncbi.nlm.nih.gov/geo/).

### 5.2. Construction of Drug Network

Drug networks were constructed using the list of drugs and the method published previously [27]. Drug targets of all the drugs were extracted from STITCH and their gene expression from CMAP. In particular, from the ranked matrix (differentially expressed genes) published on CMAP, the top 5% of over and under expressed genes were used for each drug. Previously, the drug-induced lung injury network consisted of the gene expression profiles of cancer cell lines that were downloaded from CMAP following exposure to drugs that reportedly cause treatment-related lung injury, prior knowledge of protein connectivity, and pharmacological targets of drugs [27]. Here we used the same principle, a drug’s mode of action was modeled based on the same method for drugs available in CMAP (https://www.broadinstitute.org/cmap/), and constructed the network activity of each drug.

### 5.3. Computational Identification of Potential Drugs

Once we had the network activities for the anthrax infection and for every drug in the form of two vectors containing the activities of the nodes, we computed the Euclidean distance between every drug vector and the negative anthrax vector (opposite of anthrax activity). Euclidean distance denotes the ordinary distance between two points in the Euclidean space.

The square of the Euclidean distance penalizes synergies four times as much as non-action. By synergies we mean instances where a drug enhances the anthrax effect of a particular node (inducing the same activity). The resulting scores were then divided by the number of nodes involved in the calculation to normalize across the different datasets. In this way, a drug that completely reversed the anthrax network would have a score of 0 while a drug that had no effect on the network would have a maximum score of 4. Drugs were scored based solely on the overlap of their signaling networks with the anthrax network, all the other nodes of their networks were not considered.

### 5.4. Validation

We compared the retrieved networks with the DILI network reported previously [27], calculated the overlap of our networks with the DILI nodes and computed the probability of this overlap resulting by chance, and determined whether our networks were enriched in DILI nodes.

We also searched the literature to further validate our results with a list of anthrax-treating drugs published in the literature. We mined and referenced literature reports for the drugs/compounds that have been shown to have in vitro effectiveness against anthrax or for relevant biological activities to evaluate our computational rankings of the top and bottom 10 drugs.

## Figures and Tables

**Table 1:** Lethal toxin impairs host’s innate and adaptive immune responses and damages vascular integrity and function.

Published Results	References
Studied in dendritic cells, LT (10 μg/mL, 6-h incubation) was shown to impaired adaptive immunity.	Agrawal et al. [16]
Inhibition of MAPK signaling pathways by LT impaired innate and adaptive immunity, as well as vascular barrier integrity (review).	Agrawal et al. [17]
LT (1 μg/mL, 12–72 h of incubation) induced a concentration- and time-dependent increase in vascular permeability (primary human lung microvascular endothelial cells).	Warfel et al. [24]
Studied in nonhuman primate alveolar macrophages, LT (1 μg/mL, 4-h incubation) impaired host’s innate immune responses.	Ribot et al. [15]
LT (2 μg/mL, 45-min incubation) decreased activation of p38 substrate kinase, MK2, and reduced phosphorylation of HSP27, leading to damaging endothelial barrier and vascular integrity (rat pulmonary microvascular endothelial cells).	Liu et al. [23]
Studied in PBMCs, LT (0.5 μg/mL, 15-h incubation) caused apoptosis and reduced production of pro-inflammatory cytokines.	Popova et al. [18]

**N**ote: PBMC: peripheral blood mononuclear cells; LT: lethal toxin; MAPK Mitogen-activated protein kinases; MK2: MAPK-activated protein kinase 2; HSP27: Heat shock protein 27; p38MAP kinases.

**Table 2:** Edema toxin impairs host’s innate and adaptive immune responses and damages vascular integrity and function.

Published Results	References
In human monocytes, edema toxin (20 ng/mL, 1-h incubation) induced cAMP accumulation and damaged cellular antimicrobial activity.	Hoover et al. [21]
Cooperating with lethal toxin, edema toxin impaired innate immune responses (maurine bone marrow-derived dendritic cells, 40ng/mL for both ER and LT, 1-h incubation).	Tournier et al. [19]
Studied in mice, edema toxin and lethal toxin (PA:10 μg/mL, LF and EF: 7.5 μg/mL, injection) inhibited T cell activation, implying impairment of adaptive immune response.	Comer et al. [20]
Edema toxin inhibited endothelial cell chemotaxis via Epac, the effector of RAP1 (endothelial cell line:HMVECs; PA:5 μg/mL, EF: 1 μg/mL, 1-h incubation).	Hong et al. [26]
Edema toxin (PA:5 μg/mL, EF: 1.15 μg/mL, 24-h incubation) suppresses human macrophage phagocytosis by deregulating cAMP-dependent protein kinase pathway.	Yeager et al. [22]
Edema toxin (PA:70 mg/mouse, EF: 70 mg/mouse, tail end injection) induced transendothelial cell macroaperture (TEM) tunnels (intestine) via affecting c-AMP signaling (human umbilical vein endothelial cells, 1 μg/mL ET, 1-h incubation).	Maddugoda et al. [25]

**Table 3:** The top 10 drugs out of 474 drugs/compounds ranked and their relevant biological evidences.

Drug	Biological Evidences	References
Fenofibrate (PPARα) activator	Cross talks between mevalonate pathway and PPARα; inhibition of LT cytotoxicity by statins mediated via inhibiting Rho GTPase and activating PPARα.	deCathelineau et al. [36]; Martin et al. [42]
Dihydroergotamine	5-HT 1B/1D agonist Stimulation of 5-HT 1B/1D receptors activated MAPK and reduced cAMP level.	Kayser et al. [48]; Hinton et al. [49]
Cotinine	Activated mitogen-activated protein kinases.	Warren et al. [51]; Tsai et al. [50]
Simvastatin	Statins inhibited LT cytotoxicity by inactivating Rho GTPase.	deCathelineau et al. [36]
Amantadine	Cancelled activation of p38/MAP. p38/MAP kinase inhibitors (SB-203580 and SB-202190) protected cells from LT-mediated cytotoxicity.	Eckels et al. [44]; Sanchez et al. [32]
Mephenytoin	A derivative of phenytoin. Phenytoin inhibited active transport of Ca^+2^ via enterocytes, and Ca^+2^ channel in the brain.	von Borstel Smith et al. [45]; Sitges et al. [46]
Mefloquine	Mefloquine is an analog of chloroquine that had in vitro activity protecting cells from LT toxicity.	Thompson et al. [47]
Bepridil	Calcium channel blocker; Ca^+2^ is required for LT toxicity.	Sanchez et al. [32]; Bhatnagar et al. [35]
Sotalol	Decreased intracellular accumulation of cAMP, an action that is opposite to that of ET.	Wisler et al. [53]
Ifosfamide	Increased renal recreation of Ca^+2^ that could lead to disturbance of Ca^+2^ homeostasis and depletion of Ca^+2^.	Ho et al. [52]

Note: PPARα: peroxisome proliferator receptor alpha; MAPK: mitogen-activated protein kinases.

**Table 4:** The bottom 10 ranked drugs out of 474 drugs identified.

Drug	Biological Evidences	References
Monastrol	Arresting cells in mitosis.	Cochran et al. [54]
Colforsin	An agonist of adenyl cyclase that converts ATP to cAMP. Such action would increase intracellular cAMP and synergistically increase ET toxicity.	Johannessen et al. [57]
Berberine	Reduced activation of MAPK signaling by chikungunya virus.	Varghese et al. [58]
Withaferin a	Activated p38 MAPK, a downstream kinase of MAPK signaling.	Shi et al. [59]
Arecoline	Its action is opposite to that of P38 MAPK inhibitors (Its induction of CTGF expression was inhibited by P38 MAPK inhibitors).	Deng et al. [61]
Ebselen	Inhibited ASK1-p38 MAPK-p35 and JUK signaling and activated MPAK p44/42.	Sarker et al. [55]
Genistein	Activated MAPK p44/42.	Yu et al. [56]
Apigenin	Inhibited MAPK (an action similar to LT).	Liu et al. [62]
Beclometasone	Activated p38 MAPK (an action opposite to that of p38 MAPK inhibitors in protecting cells from LT).	Boncompagni et al. [63]; Sanchez et al. [32]
Enilconazole	Antifungal drug for animals.	Merck veterinary manual

## Supplementary Materials

The following are available online at www.mdpi.com/2072-6651/9/3/99/s1, Table S1: Perturbed_genes_cell, consisting of *perturbed_genes_ham & perturbed_genes_pbmc*. For the human alveolar macrophage (HAM) dataset, there were in total 280 significantly perturbed genes (205 up- and 75 downregulated), while for the PBMC dataset there were in total 407 (309 up- and 98 downregulated), Table S2: Reactome_genes_cells.txt. The final lists for the 2 datasets used are 132 genes for the HAM dataset (101 up- and 31 downregulated) and 320 genes for PBMC (45 up- and 275 downregulated), Table S3: Inputs_cells: inputs_ham & inputs_pbmc. We used, as the starting nodes of the signal transduction (stimuli), the targets of both Lethal Toxin (LT) and of the Edema Toxin (ET), Table S4: xs_cells: xs_ham & xs_ham. The nodes included in the final network after algorithm optimization, Table S5: Results.xlsx. For each drug, we used its network activity and the anthrax activity network in the form of two vectors containing the activities of individual nodes for computation, and scored each drug using Euclidean distance.
